# DPD Testing Before Treatment With Fluoropyrimidines in the Amsterdam UMCs: An Evaluation of Current Pharmacogenetic Practice

**DOI:** 10.3389/fphar.2019.01609

**Published:** 2020-01-28

**Authors:** Forike K. Martens, Daan W. Huntjens, Tessel Rigter, Meike Bartels, Pierre M. Bet, Martina C. Cornel

**Affiliations:** ^1^Department of Clinical Genetics, Section Community Genetics, Amsterdam Public Health Research Institute, Amsterdam UMC, Vrije Universiteit Amsterdam, Amsterdam, Netherlands; ^2^Department of Clinical Pharmacology and Pharmacy, Amsterdam UMC, Vrije Universiteit Amsterdam, Amsterdam, Netherlands; ^3^Amsterdam Public Health Research Institute, Amsterdam UMC, Vrije Universiteit Amsterdam, Amsterdam, Netherlands; ^4^Department of Biological Psychology, Vrije Universiteit Amsterdam, Amsterdam, Netherlands

**Keywords:** DPYD gene polymorphism, fluoropyridine, pharmacogenomics and personalised medicine, toxicicity, electronic patient record, qualitative & quantitative analyses

## Abstract

**Introduction:**

The fluoropyrimidines (FP) (5-Fluorouracil, capecitabine, and tegafur) are commonly used anti-cancer drugs, but lead to moderate to severe toxicity in about 10–40% of patients. DPD testing [either the enzyme activity of dihydropyrimidine dehydrogenase (DPD) or the *DPYD* genotype] identifies patients at higher risk for toxicity who may be treated more safely with a lower drug dose. The Netherland's National guideline for colon carcinoma was updated in 2017 to recommend *DPYD* genotyping before treatment with FP. Pretreatment *DPYD* genotyping identifies approximately 50% of the patients that will develop severe FP toxicity. The aim of the study was to assess the uptake of DPD testing in the Amsterdam University Medical Centers over time and to evaluate stakeholder experiences to indicate barriers and facilitators of implementation in routine clinical care.

**Materials and Methods:**

We used a mixed-method approach involving electronic patient records of 753 unique patients and pharmacy information systems analyses and fifteen semi-structured interviews with oncologists, pharmacists, and patients. The constellation perspective was used to identify barriers and facilitators at the level of practice, culture and structure. The proportion of FP users who were DPD tested pretreatment showed an increase from 1% (1/86) in Q2-2017 up to 87% (73/84) in Q4-2018. Unlike a landmark paper published in 2015, the National guideline for colorectal carcinoma followed by meetings to achieve local consensus led to this steep increase in the proportion of patients tested.

**Results:**

Facilitating factors for stakeholders to implement testing included the existence of clear protocols, (anecdotal) evidence of the utility, being aware that peers are adhering to standard practice and clear and simple procedures for ordering and reporting. Main barriers included the lack of clear divisions of responsibilities, the lack of consensus on a test approach, long turn-around times and non-user-friendly IT-infrastructures. More professional education on the utility and limitations of pharmacogenetic testing was desired by most stakeholders.

**Conclusion:**

While the evidence for DPD testing was sufficient, only after the update of a National guideline and local consensus meetings the proportion of FP users that were DPD tested pretreatment rose to 87%. The implementation of personalized medicine requires stakeholders involved to attune practice, culture and structure.

## Introduction

5-Fluorouracil (5-FU), its oral prodrugs capecitabine and tegafur (fluoropyrimidines; FP) are amongst the most frequently prescribed anti-cancer drugs in the treatment of common cancer types such as colorectal, gastrointestinal, and breast cancer. A subset of patients (10–40%) treated with FP experience moderate to severe toxicities, including vomiting, diarrhea, and hand-foot syndrome (Amstutz et al., 2011).

Administered FP is primarily (> 80%) eliminated by the liver enzyme dihydropyrimidine dehydrogenase (DPD) (Thorn et al., 2011). Deficiency in DPD, prevalent in about 3–5% of the Caucasian population, results in decreased inactivation of FP and can lead to severe and fatal toxicity (van Kuilenburg et al., 2010). DPD deficiencies are often related to genetic variants in the dihydropyrimidine dehydrogenase gene (*DPYD*) (Amstutz et al., 2011). The genetic variant DPYD*2A has been long reported to result in decreased DPD enzyme activity, but more recently for other variants similar effects have been described, including the variants DPYD*13, c.2846A > T and c.1236G > A (Amstutz et al., 2009; van Kuilenburg et al., 2010; Offer et al., 2014). Carriers of one *DPYD*-mutation comprise the majority of deficient patients, and homozygous or compound heterozygous carriers occur in 0.3% (Henricks et al., 2018), leading to complete deficiency.

Although not all DPD deficiencies and FP toxicity can be explained by known genetic variants (Deenen et al., 2011) (Terrazzino et al., 2013), pretreatment testing for *DPYD* variants is a well-known strategy to detect DPD deficiencies and improve patient safety (Deenen et al., 2016). However, because not all variation in DPD enzyme activity can be explained by genetic variants, other methods such as DPD phenotyping may be used to detect decreased DPD activity (van Staveren et al., 2013). Patients with a complete or partly deficient DPD enzyme can be more safely treated with a reduced dose of FP or an alternative drug (Deenen et al., 2012). Recently, the advice to perform pretreatment DPD testing to optimize treatment efficacy and avoid adverse effects has been added to the Netherland's National guideline for colorectal carcinoma (NVMO, 2017).

According to the results of a poll conducted among Dutch internist oncologists (*n* = 208) in 2016 by the editorial board of *Medische Oncologie* (Medical Oncology), 65% of the oncologists test their patients for DPD deficiencies prior to treatment with FP. Results of this poll also showed that the main reasons DPD testing is not yet standard of care are the low prevalence of DPD deficiency (mentioned by 23% of respondents), the minimal cost-effectiveness (15%), the poor availability (4%) and other reasons (58%), such as that no quick test results are possible, the test is not validated, toxicity is not only seen among DPD deficient patients, and the incidence of DPD induced toxicity is rather low (NVMO, 2016).

The National guideline for colorectal carcinoma was updated in September 2017 to recommend *DPYD* genotyping before treatment with FP. Whether and to what extent the recommendation to prospectively execute *DPYD* genotyping is followed up in patients treated with FP is unknown.

The aim of this study was to assess the uptake of DPD testing before the use of FP in the Amsterdam University Medical Centers [UMCs, locations VU University Medical Center (VUMC) and Amsterdam Medical Center (AMC)] over time, and to evaluate stakeholder experiences to ultimately indicate barriers and facilitators of DPD testing implementation as routine clinical care. The results of this study will hopefully inform colleagues elsewhere who also strive for 100% patient safety in the end, and now are implementing DPD testing step by step. Barriers and facilitators identified in Amsterdam may apply elsewhere too.

In discussions about optimal strategies for DPD testing, our AMC colleague Van Kuilenburg (van Kuilenburg et al., 2010; van Kuilenburg et al., 2010) has been quite active in test development. As he still works on improvement of the test sensitivity, at AMC DPD phenotyping by assessing the enzyme activity is performed (**Table 1**).

**Table 1:** Specification of DPD tests used in the Amsterdam UMCs.

	VUMC	AMC
*Test*	*DPYD* genotyping	DPD phenotyping + successive genotyping for deviating enzyme activities
*Variants*	DPYD*2A (c.1905+1G > A)	Whole *DPYD* gene, including deletions and amplifications
	DPYD*13 (c.1679T > G)	
	c.2846A > T	
	c.1236G > A	

Furthermore, we apply the “constellation perspective”, by structuring the influences on implementation, as mentioned by the stakeholders, in terms of changes in culture, structure, and practice (Rigter et al., 2014). By doing so we aimed to define lessons learned for implementation of other pharmacogenetic applications beyond oncology and beyond DPD.

## Materials and Methods

### Design

A mixed methods approach was used involving patient records and pharmacy information systems analyses (quantitative analysis) and stakeholder interviews (qualitative analysis). All research was done within Amsterdam UMC, which comprises of location VUMC and AMC. This study was approved according to the national legislation. The Medical Ethical Committee of the VU University Medical Center Amsterdam evaluated the study design and decided that the Medical Research Involving Medical Subjects Act (WMO) does not apply to this study and that further official approval is not required (2019.069).

### Quantitative Analysis

The Research Data Platforms of Amsterdam UMC contain retrospective data of different software systems. For location VUMC, data was extracted *via* this platform from EPIC (electronic patient information system) and GLIMS (laboratory information system). For location AMC, also EPIC was used *via* the Research Data Platform, but laboratory information was extracted from Genesis (a clinical genetics information system).

We selected all patients that started FP treatment (Anatomical Therapeutic Chemical (ATC) codes: L01BC53, L01BC02, and L01BC06) between the 4th quarter of 2016 up to and including the 4th quarter of 2018. For all patients we collected the following data: anonymously encrypted unique patient ID, ATC codes, medication name, start/stop date of administration, dose, medication status, administration route, and DPD analysis date.

The implementation of the pretreatment *DPYD* genotyping at Amsterdam UMC location VUMC was evaluated for subsequent quarters by determining the proportion of patients who started FP treatment and were registered as DPD tested. For AMC similar calculations for DPD phenotyping were made. Patients receiving topical 5-fluorouracil (part of ATC code L01BC02) were excluded from the analysis because the guideline applies to systemic use only. Side effects are less likely for topical application. The date of the first administration was used to determine the quarter. The DPD analysis date was used to determine if DPD testing was executed. Additionally, we compared the date of the first administration with the DPD analysis date. All analyses were performed using Microsoft Excel 2016.

Several key moments in relation to the introduction of DPD testing were considered. In 2015 Meulendijks et al. published a landmark paper (Meulendijks et al., 2015). In the 3^rd^ quarter of 2017 the Netherland's National guideline for colon carcinoma was updated (NVMO, 2017). Finally, in the 4^th^ quarter of 2017 local consensus was reached to test all patients receiving FP.

### Qualitative Analysis

#### Theoretical Framework

The “constellation perspective” was applied in the development of the interview-guide, as well as the analysis of the results from the interviews (Rigter et al., 2014). This theory implies that a group of individuals or actors (professionals and patients) are used to working in a certain structure, culture, and practice (the constellation) and by this are defining and fulfilling a function in a larger societal system. As such, different ways of doing (practice), thinking (culture), and organizing (structure) by the actors are needed to achieve fundamental changes in the constellation.

#### Participants

Stakeholders were selected such that a comprehensive overview of the experiences around pretreatment DPD testing in Amsterdam UMC could be developed. Relevant stakeholders included oncologists who treated patients with colorectal, gastrointestinal, and/or breast carcinomas, hospital and outpatient pharmacists, and lab specialists involved in DPD testing at the Amsterdam UMC. DPD tested patients were identified and invited for an interview through the interviewed oncologists. In total 15 interviews were conducted in the Amsterdam UMC and outpatient pharmacies on location AMC and VUMC between February 2019 and June 2019, after which data saturation was reached. The interviews were held with 6 oncologists (2 AMC; 4 VUMC), two clinical hospital pharmacists (1 AMC; 1 VUMC), two outpatient pharmacists (1 AMC; 1 VUMC), one lab specialist (AMC), and four patients (1 AMC; 3 VUMC). All interviews were conducted face-to-face; 9 interviews by two interviewers (FM and DH) and the other 6 interviews by one interviewer (FM). Informed consent was signed by all participants before the start of the interviews.

#### Interview Guide

A semi-structured interview guide was developed based on the constellation perspective (with key concepts culture, structure, practice) and main themes from literature on barriers and facilitating factors for implementation of pharmacogenomics (Rigter et al., 2014). The guide covered the following topics for oncologists, pharmacists, and lab specialists: the current situation of *DPYD* genotyping and/or DPD phenotyping, the procedures around DPD testing, the reasons for and experiences with the current approach, and barriers and facilitating factors of implementing this test. Patients were asked about their experience and expectations about the information provision around DPD testing. The interview guides for patients and professionals are available as **Supplementary Material**. Depending on the background and expertise of the interviewee details of the interview guide have been adjusted and/or omitted.

#### Data Preparations and Analysis

Interviews were audiotaped and transcribed verbatim and content analysis was performed using Atlas.ti (Version: WIN 7.5). Transcripts were read and discussed by two researchers (FM, TR). First, recurring topics were labeled. Second, all labels were clustered based on “current practice” and the elements culture, structure, and practice of the constellation perspective in order to identify main themes. Differences in coding were discussed until consensus was reached. Representative quotes were selected and member-checked and translated into English to illustrate findings.

## Results

### Uptake of DPD Testing

**Figure 1:**
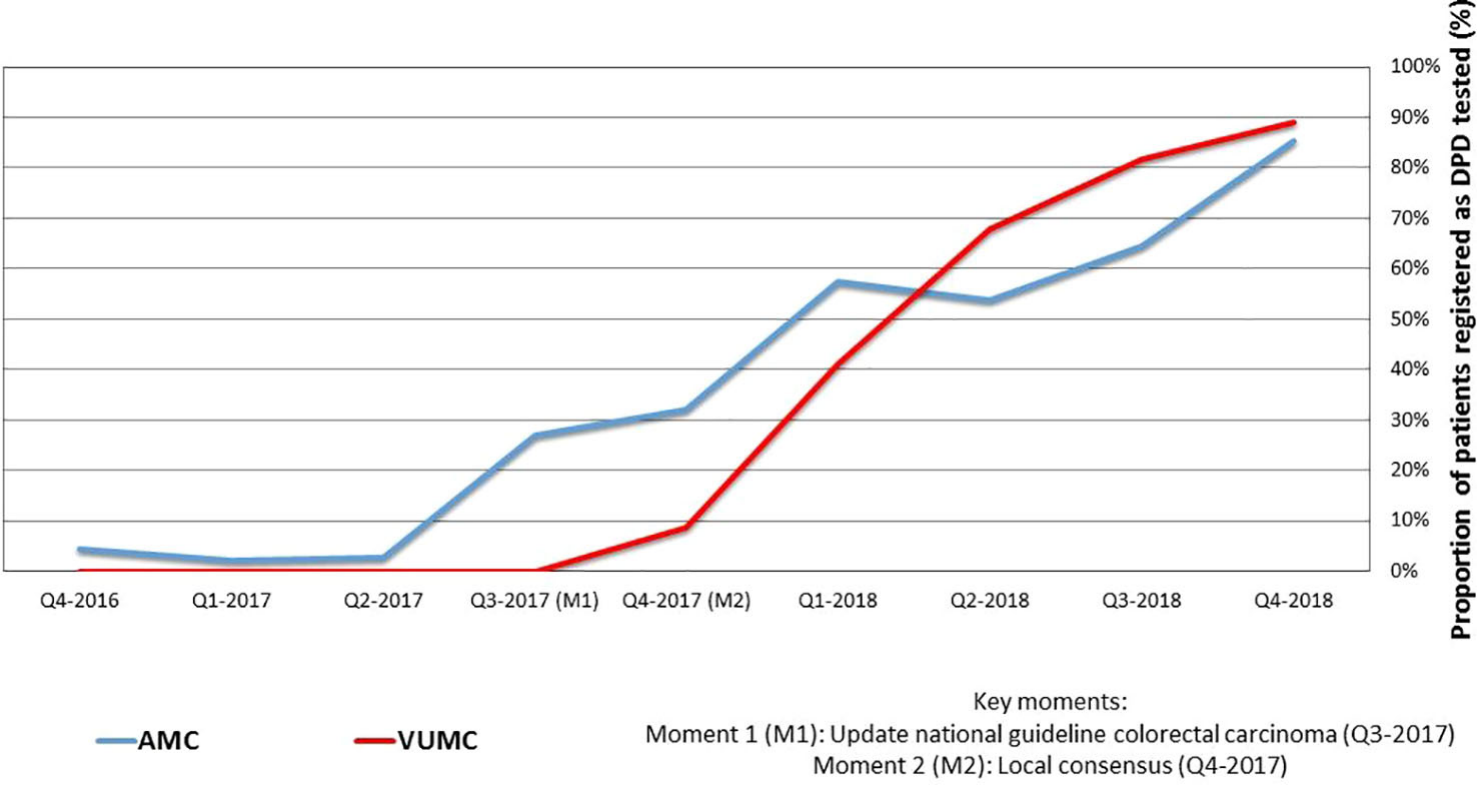
Quarterly collected proportions of patients receiving fluoropyrimidines registered as DPD tested in VUMC and AMC.

For a total number of 753 unique patients FP was prescribed and 252 patients received DPD testing. In **Table 2** the results are specified per center and quarter. **Figure 1** shows the proportional results of the quantitative analysis. The chart shows a relative increase in the proportion of DPD tested patients starting FP treatment after the 2^nd^ quarter of 2017. In Q2-2017 1/86 patients were tested. The start of the increase coincides with the updating of the National guideline for colon carcinoma and the local consensus meetings. In the 4^th^ quarter of 2018, 87% of the initiated patients (73/84) was registered as DPD tested.

**Table 2:** Number and percentage of patients using fluoropyrimidines who had been DPD-tested before the start of treatment.

Time period	DPD tested AMC				DPD tested VUMC			
	Yes	No	Total	*percentage*	Yes	No	Total	*percentage*
Q4-2016	0	37	37	*0*	2	43	45	*4*
Q1-2017	0	50	50	*0*	1	48	49	*2*
Q2-2017	0	49	49	*0*	1	37	38	*3*
Q3-2017	0	32	32	*0*	10	27	37	*27*
Q4-2017	4	42	46	*9*	15	32	47	*32*
Q1-2018	14	20	34	*41*	27	20	47	*57*
Q2-2018	21	10	31	*68*	22	19	41	*54*
Q3-2018	31	7	38	*82*	31	17	48	*65*
Q4-2018	32	4	36	*89*	41	7	48	*85*
Total	102	251	353	*29*	150	250	400	*37*

Additionally, we compared the date of administration to DPD analysis date. Ninety-one percent of patients (229 out of 252, 91%) received FP only after the result of the DPD test was known.

### Barriers and Facilitators for Implementation

The interviews with key stakeholders revealed several themes, including the needs and barriers regarding the implementation of the DPD test in clinical practice. Relevant themes are discussed below, starting with the current situation in the Amsterdam UMCs, followed by the changes that were needed to achieve the current situation and that are needed to improve implementation of pretreatment DPD testing, clustered into the three levels of the constellation perspective (practice, culture, structure).

### Clear Procedures (Practice)

#### Current Situation

In the interviews, oncologists and a pharmacist of the VUMC expressed that they follow the recommendation to conduct *DPYD* genotyping for all patients prior to receiving FP treatment.

“Yes. For everyone who will get treated with 5-FU or capecitabine we determine the DPYD gene activity.” (#6, fellow medical oncology VUMC)

The *DPYD* genotyping test on four variants (see **Table 1**) is outsourced and performed in the Erasmus Medical Center Rotterdam.

Oncologists of the AMC and a lab specialist expressed that their hospital uses a phenotypic test instead, which is performed in-house. If the results of the phenotypic test are aberrant, a successive genotyping test of the whole *DPYD* gene is performed at the VUMC.

*“*It is standard procedure that we order the DPD *[phenotypic]* test in advance, before we start with therapy*.” (#5, fellow medical oncology AMC)*

“*In our hospital, performing a DPD test before the start of the therapy is a standard procedure.”(#11, lab specialist AMC)*

Outpatient pharmacists and one hospital pharmacist of the AMC were unaware of the existence of a DPD test.

“Well, I rarely see it [DPD orders/test results]. I don't think it is standard procedure.” (#9, hospital pharmacist AMC)

“I don't know whether this is standard procedure in this hospital. We at least don't have a role in it.” (#10, outpatient pharmacist AMC)

Patients recalled that they were tested for DPD deficiency. Most of them needed some time and explanation before remembering undergoing the test.

“I can't remember exactly and I don't think it has been said emphatically. When they took a liver biopsy, they also analyzed that [DNA test]. That is when I received the gene passport, which was used to determine whether I could possibly receive different therapy. So, maybe I knew it [being tested] this way.” (#13, patient)

In general, oncologists and pharmacists did not know why either the genotyping or phenotyping DPD test was chosen.

“No, I have no idea. I have never looked into that [choice for genotyping].” (#3, oncologist VUMC)

Opinions vary about the turnaround time of the test in the VUMC, but it perceived as too long by many. Some oncologist stated that it could take up to two weeks, another mentioned half a week, another 7–8 days and a pharmacist thought it would be a maximum of three days.

The duration of the phenotyping is 4–10 days according to a lab specialist and the successive genotyping may take up to two weeks, but is often done quicker. Oncologists of the AMC experienced that it may take up to two weeks, but approximately a week to 10 days when no further genotyping needs to be done.

“Then [when no aberrant values have been found]it takes approximately a week.” (#4, oncologist AMC)

The turnaround time is seen as an important barrier by AMC oncologists.

“Yes, I think that's [waiting a week] too long.” (#4, oncologist AMC)

##### Division of Roles and Responsibilities

All interviewees expressed that oncologists, as head practitioners, have the main responsibility for initiating the DPD test.

“I have the impression that this [oncologist is responsible for having DPD test result before start] view is shared by all. I simply cannot order treatment when I haven't seen it [DPD test result] [… ].” (#2, oncologist VUMC)

According to a hospital pharmacist of the VUMC, they check whether DPD has been assayed. AMC pharmacists indicated that they are not involved in the process. Oncologists of the AMC, however, expressed that the nurses often check whether DPD results are known.

“The oncology nurses will always double check if it is safe to start with the DPD test included.” (#5, fellow medical oncology AMC)

Although outpatient pharmacists and the AMC pharmacist indicated that they are not involved, they expressed interest in the monitoring of medication and the need for clarity of the procedures and responsibilities.

“I think it is an important part of our responsibility to check this, yes.” (#7, outpatient pharmacist VUMC)

“Legally this is correct. In practice, both doctors as well as pharmacists and other healthcare providers are responsible for doing something with aberrant lab values or results, but this must always happen in full agreement between professionals. In the outpatient pharmacy we work with 2 systems (AIS CGM Pharmacy and ZIS EPIC), which makes it more complicated to properly check and record data as well as any follow up actions.” (#10, outpatient pharmacist AMC)

“[… ], so I think it is important that we are getting involved with the implementation of such a project [DPD testing for patient receiving fluoropyrimidines] and if that hasn't been the case, then it's a bit disappointing.” (#7, outpatient pharmacist VUMC)

##### Communication Is Key

Oncologists and a lab specialist are not aware whether and in what way the pharmacists are involved and have generally no contact with each other. Only some oncologist indicated to have contact about logistics or to answer questions concerning adjusting the treatment dose. Hospital pharmacists are unaware of any involvement of the outpatient pharmacists and vice versa.

“There might be some uncertainty. [… ] Most of the time it [capecitabine] is provided by the outpatient pharmacy. I don't know whether they perform a check” (#8, hospital pharmacist VUMC)

##### Information Provision to Patients

All oncologists expressed that they informed their patients about the DPD test, most of them prior to the start of the therapy. The communication included information on why the test was done and if applicable how the dose of medication was reduced. One oncologist indicated that patients are only informed more extensively when the test results are aberrant.

“We communicate more extensively when it [DPD test result] is aberrant and we will give a lower dose of chemotherapy. [… ] When it [test results] is okay, I inform the patient that the DPD results are good and no adjustment of the therapy needs to be made.” (#2, oncologist VUMC)

According to the patients the information provision was sufficient. Patients for whom the start of the therapy was longer ago did not remember exactly when the information was provided.

“Yes, she told me [enough information].” (#14, patient)

One patient who recently started therapy added that, although the information was sufficient, a simpler (in layman's terms) explanation of what the DPD test exactly is would have been favorable.

#### Convincing Stakeholders of Need and Utility (Culture)

The main themes identified as important for changing culture regarding pretreatment DPD testing were evidence (scientific and anecdotal), willingness to follow guidelines, shared views with coworkers and the perceived need to start treatment as quickly as possible after diagnosis.

##### Evidence For Clinical Utility/Usefulness

In the guideline *DPYD* genotyping is advised. Most participants were convinced of the importance of this test in order to prevent toxicity and FP related death. However, in general interviewees had no clear idea why the current approach (genotyping or phenotyping) was chosen.

In general, VUMC oncologists indicated that the test is clinically useful, however, some oncologists questioned the need and clinical utility of the test as they mentioned that the occurrence of toxicity due to DPD deficiency is low and the test not able to perform 100% accurately.

“Look, I think you have to test many people to really significantly reduce morbidity or mortality. It is worth a lot to prevent every death, that's the truth of course, but we actually encountered severe toxicity once a year at most. Very few. [… ] but recently there have been publications on routine screening to prevent toxicity, so there is [some] more evidence.” (#1, oncologist VUMC)

“Well, I have always heard that it wasn't a good test and that you will still not find 50% of the people [with a DPD deficiency], or something like that.” (#2, oncologist VUMC)

Another oncologist expressed the importance of having a realistic understanding of the limitations of the test.

“Yes, I think people are pretty aware right now, but I can imagine that in the future the story will be; you can have toxicity, but when you do that test then you won't have it. So I think it is very important to keep saying that this is only a small part of the possible gene abnormalities and apart from genetic [causes], you can also have toxicity due to other reasons.” (#6, fellow medical oncology VUMC)

Also oncologists of the AMC expressed that they were convinced of the clinical usefulness of the DPD test.

“The DPD deficiency is proven by science and is very important, so we can treat our patients safe.” (#5, fellow medical oncology AMC)

However, one of them questions whether the current approach in AMC is evidence-based.

“Well a barrier for me, or not particularly a barrier, but more a doubt why or to what extent it is evidence-based what we do with the phenotyping. Everyone knows about the evidence of cost effectiveness of the genotyping [4 variants], but we phenotype. So, that I find a bit hard.” (#4, oncologist AMC)

Pharmacists are enthusiastic about DPD testing, even when they indicated that it is not yet standard procedure. One hospital pharmacist expressed that besides guidelines, the scientific evidence for DPD testing is important.

“But when there is scientific evidence and guidelines state that the pharmacy needs to monitor it, then I will definitely do so.” (#9, hospital pharmacist AMC)

A lab specialist expresses that in order to provide the best possible patient care, not costs but rather the effectiveness of the test [in preventing toxicity] should be leading in future decisions about which DPD test (genotyping/phenotyping) to use.

“Having a DPD deficiency is a contraindication for being treated with 5-FU or capecitabine. So, providing a patient a suboptimal test, when knowing there is a better test, I think one doesn't act ethically.[… ] So, I understand the [need for evidence on] cost effectiveness, but I also think it gets a bit exaggerated sometimes.” (#11, lab specialist AMC)

##### Experiences With Relevant Cases

An experience with an aberrant genotype that potentially would have been missed with the current DNA test was described by a lab specialist:

“That was a patient who was diagnosed by a hospital as a carrier of one of the four pathogenic variants [c.1905+1G > A] in the DPD gene. Fortunately, they also sent us a blood sample and we actually found a complete DPD deficiency when we analyzed the DPD enzyme activity. When we performed an extensive analysis of the DPD gene (DPYD) we discovered that the patient was heterozygous for an amplification of part of the DPYD gene. Such an amplification is very rare and this patient was eventually treated with only 0.8% of the normal dose and would have died if treated with 50% of the normal dose, which is the recommended dose for carriers of this particular variant”.(#11, lab specialist AMC)

##### Adhering Guidelines

In general, oncologists expressed that they simply follow the guideline. However, they also indicated that prior to the implementation of the DPD test they were already able to monitor patients adequately. They expressed that the DPD test is a helpful tool, but remaining critical on what is best for the patient is important.

“I think it can contribute, but may also give false security [… ] I don't see it as a holy grail.” (#6, fellow medical oncology VUMC)

“[… ] before [implementation of the DPD test] we haven't done it for very long. Back then we dosed on the basis of how the patient was doing in the first weeks, and since it is not that prevalent, there is something to say for that as well [… ]. On the other hand, the impact of DPD deficiency can be huge, with serious morbidity and mortality that can be avoided by a relatively simple DPD test.” (#3, oncologist VUMC)

However, they do not always follow the protocol. According to an oncologist (VUMC) they do not order *DPYD* genotyping, when patients have been treated successfully during a previous treatment cycle of 5 FU.

“Then we have proof that it is well tolerated.” (#2, oncologist VUMC)

##### Starting Before Results

Many oncologists indicated that waiting for test results could take (too) long, which causes an unnecessary delay. As a solution they mentioned that they start treatment on a lower dose, before test results are available.

“The DPD test will take 2–3 weeks to be known. If it is necessary we will start our therapy 50% lower dose until the results are to be known.” (#5, fellow medical oncology AMC)

“Certainly, when it causes a hassle and when you have to postpone, then you think I'll just start and will raise the dose later, or I call [the patient] after three days and then I'll still be able to reduce the pills you know, they take it every day, you have some room to play a bit.” (#6, fellow medical oncology VUMC)

##### Shared Views With Peers And the Need for a Convinced Supervisor

A facilitating factor to implement DPD genotyping is that views were shared with their co-workers, according to the interviewees.

“Well, in the same way. Everyone is convinced I think, that one way or the other you have to test on such a lowered function of the DPD enzyme and whether that is genetic or phenotypic, that doesn't really matter. [… ], but everyone is convinced that a test has to be performed I think.” (#4, oncologist AMC)

“Yes, they[my colleagues] support that.” (#11, lab specialist AMC)

One oncologist indicated that a possible reason for why DPD testing was not part of standard procedures before the update of the guideline in 2017, was the fact, that the lead oncologist lacked personal belief in the added value of DPD testing.

“I think it is the policy of the head of the department, whom is then not convinced.” (#2, oncologist VUMC)

#### Facilitating Safe and Effective Procedures (Structure)

The three main themes identified as important for changing structure were: logistics (process automation)/infrastructure, protocols and education.

##### Logistics and Infrastructure

Most oncologist of the VUMC indicated that the process of ordering a DPD test is cumbersome and hence expressed that the non-automated process is a disadvantage.

“A very inconvenient method; with a lab form from the Erasmus [Medical Center, Rotterdam] that we fill out by hand and sent with the patient to the blood test [facility], which will then be sent [to the Erasmus]. (#2, oncologist VUMC)

“[… ], I think actually the fact that I have to print out and then fill out, that I actually find that the most annoying.” (#3, oncologist VUMC)

AMC oncologists indicated that the DPD test is ordered digitally in EPIC (hospital information system) and expressed that they do not see any logistic barriers. Pharmacists perceived the lack of a link between several software-systems as a barrier for implementing the test and further implementation of pharmacogenetic testing and expressed that a prerequisite for proper functioning of the process is that all systems communicate.

“Definitely, but a barrier is that not all support and systems work optimal. [… ] Logistics are kind of a challenge and that may be why it [DNA medication pass] isn't used that much. [… ] So, I think it [optimal systems and support] is a prerequisite for how this will work.” (#9, hospital pharmacist AMC)

Pharmacists also expressed that only ten contraindications can be registered in the outpatient pharmacy system, which requires expansion when more pharmacogenetic tests will be performed.

### Protocols

A main prerequisite for implementation of the DPD test are the protocols and agreements. Most oncologist expressed that they perform the test, because it is the protocol.

“[… ] Since we have made the decision to not start before [having a DPD test result], we adhere to this.” (#2, oncologist VUMC)

“Yes, it is an obligation. So it is seen as a fault to not test.” (#2, oncologist VUMC)

“Yes. Before [the guideline update] we did not do that [DPD genotyping], but since the recommendation has been included in the guideline, we adhere to it.” (#1, oncologist VUMC)

This opinion is also shared by a lab specialist, who indicated that including the instruction to perform DPD testing in a protocol promotes compliance.

“I think that in general healthcare is very protocol driven, so when something is not in a protocol, you will not see changes so quickly.” (#11, lab specialist AMC)

The reports with the test results were perceived as adequate and stakeholders indicated to have enough knowledge to change the treatment dose appropriately.

“That [the dose advice] is included [in the VUMC report], we don't need to look it up.” (#3, oncologist VUMC)

However, one oncologist of the AMC acknowledged that it would be of great support when the test result report could include instructions on how to interpret the test results.

“Well, no. I often have to call, because it is unclear to me. So, yes, I think this could be better. I think when you have results that state it is normal or reduced that it also immediately says, in this range the advice is to start with this much of a percentage of the normal dose. That is not included.” (#4, oncologist AMC)

### Education

Generally, oncologists and pharmacists indicated that knowledge was sufficient. However, some mentioned more professional education is needed; that education could possibly benefit doctors in training.

“Yes [education is a prerequisite], especially when we will be involved.” (#9, hospital pharmacist AMC)

“Well, it is, I think for people in training it will be good to know why we do it.” (#3, oncologist VUMC)

## Discussion

Over a two-and-a-quarter years' time period, 753 patients started FP treatment at Amsterdam UMC. The proportion that was DPD tested before the start of the treatment started to increase around the time of the publication of National guideline for colorectal carcinoma, which was also discussed at local meetings to achieve consensus between oncologists and pharmacists at a local level. The publication of a landmark paper two years before had no effect in terms of implementation. The increase of the proportion of patients tested continued to the fourth quarter of 2018, when 87% was achieved. Guidelines clearly are very important for implementation, as well as multidisciplinary local meetings to achieve consensus at a local level.

According to our data, around 13% of patients were not tested against the end of the study period. Perhaps some of these had received FP treatment before, without experiencing side effects, or had been tested in other hospitals. The possibility, however, that DPD-testing could have been “forgotten” for some patients led to renewed discussions in 2019. Since the goal is to achieve 100% pretreatment DPD testing in order to maximize patient safety, additional checks have been built recently in the medical protocols, the electronic ordering system, and dispensing protocol by both the clinical as well as the out-patient pharmacists. Protocols for these different sites were attuned. Also on the oncology wards, nurses started to check DPD status as part of their standard protocol.

The 2017 National guideline applies to colon cancer, but apparently the uptake of DPD testing increased overall. From a biological point of view evidently similar toxicity is at stake. From an implementation point of view it is remarkable to see that a protocol in one field may stimulate implementation of innovation overall.

Since the update of the guideline to conduct *DPYD* genotyping for all patients prior to receiving FP treatment, in the VUMC the patients are tested for 4 genetic variants of the *DPYD* gene, while at the AMC a conscious decision to use a phenotypic test first was made, followed by genotyping for aberrant results.

Since genotyping of a limited number of *DPYD*-variants explains only part of the variance, potentially the sensitivity of the test could be improved with phenotyping. In theory the use of assays that determine enzyme activity first, could be more predictive.

Although no agreement exists on the best test-approach, in general stakeholders are convinced of the clinical utility of DPD testing prior to FP treatment. Multiple stakeholders seem to realize that cost-effectiveness for genotyping is demonstrated, but some are convinced that phenotyping first is a better (more sensitive) method. A large prospective head-to-head comparison would be needed to identify the optimal algorithm, either one assay or a combination. Studies comparing (cost-)effectiveness of different approaches therefore seem to be warranted.

Another pressing issue that arose from the interviews was the need for a more clear division of responsibilities. Although, when asked, most stakeholders expressed that it was the oncologist's responsibility that the test was performed, no clear division of roles seemed to have been agreed upon. Especially the role of the (outpatient) pharmacist could be more formalized, at least as having a responsibility to check whether standard procedures have been followed to ensure drug safety: in this case preventing toxicity in patients with potential aberrant DPD geno- and/or phenotypes.

Patients appreciated being tested for *DPYD*-variants for reasons of medication safety. They mentioned that information in simple language was needed. For the patients it was not relevant whether or not the assay was a DNA test. They liked the idea of having possession of their own DNA test results, as well as data sharing of these results between health care professionals.

In general, facilitating factors for stakeholders to implement pretreatment testing included the existence of clear protocols, (anecdotal) evidence of the utility, being aware that peers are adhering to standard practice and clear and simple procedures. Main barriers included the lack of clear divisions of responsibilities, the lack of consensus on a test approach, long turn-around times and non-user-friendly IT-infrastructures. More education about the utility of pharmacogenetic testing, but also the limitations of such tests was desired by most stakeholders.

While we describe the situation in Amsterdam UMC only, the process we undertook to study the ongoing implementation of DPD testing before FP treatment can hopefully inspire others. While competencies required by pharmacists and other health care professionals have often mentioned knowledge and academic skills, we here illustrate the importance of the successful integration of pharmacogenomics into health and public policy. Training efforts should also include the development of implementation skills. Should other researchers repeat this study, we hope that more than 87% pretreatment testing is found, since we strive for 100% patient safety. The barriers and facilitators that we identified can hopefully contribute to optimal implementation.

### Strengths and Limitations

The study reports data from two Amsterdam University Medical Centers only. Whether the proportion of patients who have been DPD tested before the start of FP treatment increased to the same extent in other centers needs to be investigated. Local lessons on barriers for implementation, however, can inform other centers on the implementation of DPD and other pharmacogenetic tests. We have also shown that more clarity can be achieved on roles and responsibilities, to achieve optimal patient safety.

### Future Perspectives

Personalized medicine is gaining ground. In terms of implementing new tests to give the right dosage of the right medication to the right person at the right time, it is needed to have clear evidence, professional guidelines, local consensus on the practical implications of guidelines and a clear division of roles and responsibilities. Patients want to be informed about pharmacogenetic testing in simple wording. Research has to show the pros and cons of genotyping vs. phenotyping after which the two locations of Amsterdam UMC will choose one approach. The evaluation of the test has to take both test properties (sensitivity, predictive value) and cost-effectiveness into account. DPD testing is an opportunity to improve patient safety.

## Data Availability Statement

The datasets generated for this study are available on request to the corresponding author.

## Author Contributions

PB, MC and MB developed the project, FM and DH performed interviews, TR provided methodological input and analysed the interviews, DH took care of the quantitative analyses. All authors discussed and reviewed the draft versions of the manuscript and agreed on the final text.

## Funding

The study was funded by the Personalized Medicine program of the Amsterdam Public Health Research Institute.

## Conflict of Interest

The authors declare that the research was conducted in the absence of any commercial or financial relationships that could be construed as a potential conflict of interest.

## References

[B6] Amstutz U. Farese S. Aebi S. Largiader C. R. (2009). Dihydropyrimidine dehydrogenase gene variation and severe 5-fluorouracil toxicity: a haplotype assessment. Pharmacogenomics 10 (6), 931–944. 10.2217/pgs.09.28 19530960

[B1] Amstutz U. Froehlich T. K. Largiader C. R. (2011). Dihydropyrimidine dehydrogenase gene as a major predictor of severe 5-fluorouracil toxicity. Pharmacogenomics 12 (9), 1321–1336. 10.2217/pgs.11.72 21919607

[B8] Deenen M. J. Cats A. Sechterberger M. K. Severens J. L. Smits P. H. M. Bakker R. (2011). Safety, pharmacokinetics (PK), and cost-effectiveness of upfront genotyping of DPYD in fluoropyrimidine therapy. J. Clin. Oncol. 29, 3606. 10.1200/jco.2011.29.15_suppl.3606 26573078

[B12] Deenen M. J. Cats A. Mandigers C. M. Soesan M. Terpstra W. E. Beijnen J. H. (2012). Prevention of severe toxicity from capecitabine, 5-fluorouracil and tegafur by screening for DPD-deficiency. Ned. tijdschr. voor geneeskd. 156 (48), A4934. 23191966

[B10] Deenen M. J. Meulendijks D. Cats A. Sechterberger M. K. Severens J. L. Boot H. (2016). Upfront genotyping of DPYD*2A to individualize fluoropyrimidine therapy: A Safety and Cost Analysis. J. Clin. Oncol. Off. J. Am. Soc. Clin. Oncol. 34 (3), 227–234. 10.1200/JCO.2015.63.1325 26573078

[B7] Henricks L. M. Lunenburg C. de Man F. M. Meulendijks D. Frederix G. W. J. Kienhuis E. (2018). DPYD genotype-guided dose individualisation of fluoropyrimidine therapy in patients with cancer: a prospective safety analysis. Lancet Oncol. 19 (11), 1459–1467. 10.1016/S1470-2045(18)30686-7 30348537

[B16] Meulendijks D. Henricks L. M. Sonke G. S. Deenen M. J. Froehlich T. K. Amstutz U. (2015). Clinical relevance of DPYD variants c.1679T > G, c.1236G > A/HapB3, and c.1601G > A as predictors of severe fluoropyrimidine-associated toxicity: a systematic review and meta-analysis of individual patient data. Lancet Oncol. 16 (16), 1639–1650. 10.1016/S1470-2045(15)00286-7 26603945

[B14] NVMO (2016). Ernstige Toxiciteit Bij Behandeling Fluoropyrimidines Voorkomen. Medische Oncologie 2016, 12–15.

[B13] NVMO (2017). Update richtlijn colorectaal carcinoom Medische Oncologie, (21–9–2017.) [Available from: https://www.nvmo.org/wp-content/uploads/2018/07/Update_richtlijn_CRC_sept_2017.pdf

[B4] Offer S. M. Fossum C. C. Wegner N. J. Stuflesser A. J. Butterfield G. L. Diasio R. B. (2014). Comparative functional analysis of DPYD variants of potential clinical relevance to dihydropyrimidine dehydrogenase activity. Cancer Res. 74 (9), 2545–2554. 10.1158/0008-5472.CAN-13-2482 24648345 PMC4012613

[B15] Rigter T. Henneman L. Broerse J. E. Shepherd M. Blanco I. Kristoffersson U. (2014). Developing a framework for implementation of genetic services: learning from examples of testing for monogenic forms of common diseases. J. Community Genet. 5 (4), 337–347. 10.1007/s12687-014-0189-x 24895224 PMC4159469

[B9] Terrazzino S. Cargnin S. Del Re M. Danesi R. Canonico P. L. Genazzani A. A. (2013). DPYD IVS14+1G > A and 2846A > T genotyping for the prediction of severe fluoropyrimidine-related toxicity: a meta-analysis. Pharmacogenomics 14 (11), 1255–1272. 10.2217/pgs.13.116 23930673

[B2] Thorn C. F. Marsh S. Carrillo M. W. McLeod H. L. Klein T. E. Altman R. B. (2011). PharmGKB summary: fluoropyrimidine pathways. Pharmacogenet. Genomics 21 (4), 237–242. 10.1097/FPC.0b013e32833c6107 20601926 PMC3098754

[B3] van Kuilenburg A. B. Dobritzsch D. Meijer J. Meinsma R. Benoist J. F. Assmann B. (2010). Dihydropyrimidinase deficiency: Phenotype, genotype and structural consequences in 17 patients. Biochim. Biophys. Acta 1802 (7–8), 639–648. 10.1016/j.bbadis.2010.03.013 20362666

[B5] van Kuilenburg A. B. Meijer J. Mul A. N. Meinsma R. Schmid V. Dobritzsch D. (2010). Intragenic deletions and a deep intronic mutation affecting pre-mRNA splicing in the dihydropyrimidine dehydrogenase gene as novel mechanisms causing 5-fluorouracil toxicity. Hum. Genet. 128 (5), 529–538. 10.1007/s00439-010-0879-3 20803296 PMC2955237

[B11] van Staveren M. C. Guchelaar H. J. van Kuilenburg A. B. Gelderblom H. Maring J. G. (2013). Evaluation of predictive tests for screening for dihydropyrimidine dehydrogenase deficiency. Pharmacogenomics J. 13, 389–395. 10.1038/tpj.2013.25 23856855

