# Supplementary material for: DPD Testing Before Treatment With Fluoropyrimidines in the Amsterdam UMCs: An Evaluation of Current Pharmacogenetic Practice
Source: Front Pharmacol. 2020 Jan 28;10:1609. doi: 10.3389/fphar.2019.01609 (PMC6997151; doi:10.3389/fphar.2019.01609)
Supplement: Supplementary file 1 [file DataSheet_1.pdf]

**<u>Interview guide professionals</u>**

**DPD-pilot Amsterdam UMC**

The interview contains six parts:

1) Summary of study and interview
2) Informed consent
3) Reasons for/against requesting DPD-genotyping
4) Elucidating the process from test-request to communicating the results
5) Arguments for current approach
6) Wrap-up

## 1) Summary of study and interview

The study

The national guideline for colon-carcinoma is advising to conduct DPD-genotyping before treatment with fluoropyrimidines to reduce the number of cases with serious FP-induced toxicity. This interview study is aimed at health care professionals who are involved in treatment of patients with fluoropyrimidines. With approval of the Medical Ethical Committee of the VU University Medical Center you are invited for a personal interview with me, the executing researcher.

Goal of the study

With this study we would like to evaluate what considerations and experiences are relevant around DPD-genotyping. We study how often and why genotyping is requested and whether dosing is adjusted accordingly. Furthermore, we evaluate how this information is registered in the electronic patient record and the IT-system of the pharmacy and what experiences and expectations different stakeholders (professionals and patients) have considering the use of DPD-genotyping. With the results from the interviews barriers and facilitating factors for implementation can be described and implementation can be optimized.

Privacy

All data that will be obtained during the interview, will be handled confidentially. The data will be coded and processed anonymously into a research report and will not be available for (of fed back to) your superior. Your name and other personal details will not be incorporated in the report. During the interview audio recordings will be made. These will be destroyed as soon as the study is completed.

## 2) Informed consent

- Before we start I would like to ask you to fill out this informed consent form. Please read the form and if you agree with the terms you can sign at the bottom. By signing

you agree to have understood all aspects and you are willing to participate in our study.
- This interview is expected to take around half an hour. If you have any further questions or remarks you can e-mail us afterwards. The e-mail-address is on the informed consent form.

*The informed consent form is signed twice: the participant and the interviewer will both save a copy.*

- Do you have any further questions before we start?
- I will start the audio recording

**3) Current situation (whether or not) requesting according to protocol**

1a) Is it standard procedure in your center to conduct DPD genotyping/phenotyping when prescribing 5FU/capacitabine/tegafur?

1b) For which patients/under which circumstances?

- According to protocols?
- In certain study populations?
- Only after toxicity occurs?
- Only with adjuvant treatments/high dosages?

**4) The process**

2) Who initiates, in general, the request (pharmacist, physician, lab)?

3) How is the request made (standard (digital) form available, who requests who)?

4) How is the result communicated between health care professionals (time, who to who, how, text/description (e.g. including dosing advise)?

5) Are the result and dosing adjustment communicated to the patient? And how (elaborate)?

Extra questions for pharmacists:

a. Is the DPD result known in the pharmacy and is a dosing-check performed?
b. Is the pharmacist also performing dosing-checks with the results from the renal function test?

**5) Arguments current approach**

6) What are the reasons for choosing the current approach (whether or not to genotype/phenotype, or combination)?

<u>Barriers</u>

7) What is the reason not to test (all patients with 5FU treatment) standardly? What were the reasons standard testing had not been implemented previously?

- Are you personally convinced of the utility of the test (and Cost-effectiveness? Clinical validity? Prevalence DPD-deficiency? Does screening predict DPD-deficiency well enough? Many toxicity in patients without DPD-deficiency?)?

- Are you colleagues convinced of the utility of the test?

- Are you convinced that you can make decisions on adjusting dosing that are safe and still effective (are you worried about 'undertreatment'?)?

- Do you feel DPD-genotyping is the best way to determine DPD deficiency?

- Is everyone aware of the possibility of testing and how did this happen/what can still be improved?

- Are roles and responsibilities clearly allocated? (Communication between physicians/lab/pharmacists, who requests tests etc.)?

- Are there any logistical constraints (like lack of lab facilities, availability of the test, quick results of the test)?

- Are there any other reasons why testing is not standard procedure?

<u>Facilitating factors</u>

8) What would be needed to make DPD genotyping standard procedure? / How did DPD-genotyping become standard procedure for all patients who are prescribed 5FU?

- Is there a clearer distribution of responsibilities needed?

- Do you think more close collaboration between stakeholders is needed?

- Would more clear guidelines/protocols be desirable?

- Is more education needed (to whom)?

- Would more scientific evidence be needed?

- Are there any other factors relevant?

- What is your view on the potential of this test? Implementation in the rest of the Netherlands?

Other questions:

- We will process the input from this interview and will analyze it together with the data from the interviews with all stakeholders. We are thinking of physicians, lab specialist, pharmacists and patients as the most relevant stakeholders. Are there any other stakeholders that we should include?
- If interviewee does not provide standard testing: is there a colleague who does?

- Would you be prepared to recruit some patients for our study?

**5) Thank you and wrap-up**

Thank you for your participation.

- Do you have any questions?
- Would you like to add anything else?
- Did I forget to address anything relevant?

*A summary of the interview will be written with a short impression of the interview. After this the conversation is anonymously transcribed and the audio recording will be deleted. The transcript is sent to the interviewee for 'member-checking'. Furthermore, selected quotes will be translated to be included in a manuscript with the results of the study. Check for correct interpretation and specific consent for use of the quotes will be requested from the interviewee before submission to a journal.*
