# Supplementary material for: DPD Testing Before Treatment With Fluoropyrimidines in the Amsterdam UMCs: An Evaluation of Current Pharmacogenetic Practice
Source: Front Pharmacol. 2020 Jan 28;10:1609. doi: 10.3389/fphar.2019.01609 (PMC6997151; doi:10.3389/fphar.2019.01609)
Supplement: Supplementary file 2 [file DataSheet_2.pdf]

<u>Interview guide patients</u>

DPD-pilot Amsterdam UMC

The interview contains four parts:

1) Summary of study and interview
2) Informed consent
3) Information provision DNA test
4) Wrap-up

1) Summary of study and interview

The study

Since a couple of years it is advised to conduct a DNA test before treatment with chemotherapy to reduce the number of individuals with serious side-effects. This interview study is aimed at health care professionals who are involved in treatment of patients with chemotherapy and at patients whom are tested for the presence of the 'DPD gene'. With approval of the Medical Ethical Committee of the VU University Medical Center you are invited for a personal interview with me, the executing researcher.

Goal of the study

With this study we would like to evaluate what considerations and experiences are relevant around the DNA test. We study how often and why genotyping is requested and whether dosing is adjusted accordingly. Furthermore, we evaluate how this information is registered in the electronic patient record and the IT-system of the pharmacy and what experiences and expectations different stakeholders (professionals and patients) have considering the use of such DNA tests. With the results from the interviews implementation can be optimized.

Privacy

All data that will be obtained during the interview, will be handled confidentially. The data will be coded and processed anonymously into a research report and will not be available for (of fed back to) your physician. Your name and other personal details will not be incorporated in the report. During the interview audio recordings will be made. These will be destroyed as soon as the study is completed. I want to stress the fact that I am an independent researcher who has no access to your medical record. What you share with me is all I know.

2) Informed consent

- Before we start I would like to ask you to fill in this informed consent form. Please read the form and if you agree with the terms you can sign at the bottom. By signing you agree to have understood all aspects and you are willing to participate in our study.

- This interview is expected to take around half an hour. If you have any further questions or remarks you e-mail us afterwards. The e-mail-address is on the informed consent form.

The informed consent form is signed twice: the participant and the interviewer will both save a copy.

- Do you have any further questions before we start?
- I will start the audio recording

3) Information provision DNA test

1) Were you informed about (the possibility of) the DNA test to estimate your risk of severe adverse events of the chemotherapy?
2) At what point in the process did you receive this information? From whom?
3) What information did you receive?
4) Have you read about ‘pharmacogenetic testing’ in the patient information leaflet of the therapy? If yes, what did you understand from this?
5) Was the information provision sufficient? Or would you have appreciated more/less information?

In the background information it is described how several hospitals provide people with the opportunity to have a personal ‘DNA medication card’ made. With a blood or saliva test insight in the most relevant pieces of DNA that influence the breakdown of certain therapies can be retrieved. The test result is printed on a card. In order to take into account this information when prescribing therapy, your pharmacist can enter this information in his system.

6) What would you think of having such a card in the future?

(The information retrieved by this last question has not been analyzed for this study)

5) Thank you and wrap-up

Thank you for your participation.

- Do you have any questions?
- Would you like to add anything else?
- Did I forget to address anything relevant?
